# Significance of Biogenetic Markers in Giant Cell Tumor Differentiation and Prognosis: A Narrative Review

**DOI:** 10.3390/diagnostics15010039

**Published:** 2024-12-27

**Authors:** Muhammad Taqi, Haseeb ul Rasool, Mobeen Zaka Haider, Munjed Al Muderis

**Affiliations:** 1 Orthopedic Surgery, Macquarie University Hospital, Sydney, NSW 2113, Australia; 2 Internal Medicine Department, Icahn School of Medicine Mount Sinai, New York, NY 10029, USA; 3 Internal Medicine Department, Carle Foundation Hospital, Urbana, IL 61801, USA

**Keywords:** biogenetic markers of GCT, giant cell tumor, prognostic markers for GCT

## Abstract

**Background**: Giant cell tumor of bone (GCTB) is a locally aggressive tumor. It accounts for only 5% of all bony tumors. Early diagnosis, and follow-up for recurrence is often difficult due to a lack of biogenetic markers. Giant cells are multinucleated epithelioid cells derived from macrophages. Histologically, giant cells are also present in other pathologies of bone, e.g., aneurysmal bone cyst, chondroblastoma, giant cell granuloma, and malignant giant cell tumor, etc. Similarly, radiographic findings overlap with other osteolytic lesions, making the diagnosis and prognosis of giant cell tumor very challenging. **Aims and Objectives**: The purpose of this study was to explore biological and genetic markers which can be used for detection, differentiation, recurrence, and prognosis of GCTB. This will help to better understand the clinical outcome of GCTB and minimize the need for interventions. **Methods**: We conducted a literature search using Google, Google Scholar, PubMed, Wiley Library, Medline, Clinical trials.org, and Web of Science. Our search strategy included MeSH terms and key words for giant cell tumor and biogenetic markers from date of inception to September 2020. After excluding review articles, 246 duplicates, and non-relevant articles, we included 24 articles out of 1568 articles, summarizing the role of biogenetic markers in the prognosis of GCT. **Results**: P63 is 98.6% sensitive and relatively specific for GCT as compared to other multinucleated giant cells containing neoplasms. MDM2 (mouse double minute 2 homolog), IGF1 (insulin-like growth factor 1), STAT1 (signal transducer and activator of transcription 1), and RAC1 (Ras-related C3 botulinum toxin substrate 1) are associated with GCTB recurrence, and might serve as biomarkers for it. Increased expression of the proteins STAT5B, GRB2, and OXSR1 was related to a higher probability of metastasis. H3F3A and H3F3B mutation analysis appears to be a highly specific, although less sensitive, diagnostic tool for the distinction of giant cell tumor of bone (GCTB) and chondroblastoma from other giant cell-containing tumors. A neutrophil to lymphocyte ratio (NLR) > 2.70, platelet to lymphocyte ratio (PLR) > 215.80, lymphocyte to monocyte ratio (LMR) ≤ 2.80, and albumin to globulin ratio (AGR) < 1.50 were significantly associated with decreased disease-free survival (DFS) (*p* < 0.05). Large amounts of osteoclast-related mRNA (cathepsin K, tartrate-resistant acid phosphatase, and matrix metalloproteinase9) in GCTs (*p* < 0.05) are associated with the grade of bone resorption. We propose that subarticular primary malignant bone sarcomas with H3.3 mutations represent true malignant GCTB, even in the absence of a benign GCTB component. IMP3 and IGF2 might be potential biomarkers for GCT of the spine in regulating the angiogenesis of giant cell tumor of bone and predicting patients’ prognosis. **Conclusions**: This review study shows serological markers, genetic factors, cell membrane receptor markers, predictive markers for malignancy, and prognostic protein markers which are highly sensitive for GCT and relatively specific for giant cell tumor. MDM2, IGF1, STAT1, RAC1 are important makers in determining recurrence, while P63 and H3F3A differentiate GCT from other giant cell-containing tumors. STAT5B, GRB2, and OXSR1 are significant in determining the prognosis of GCT. Apart from using radiological and histological parameters, we can add them to tumor work-up for definitive diagnosis and prognosis.

## 1. Introduction

Giant cell tumor of bone (GCTB) is a locally aggressive tumor, comprising macrophage-like cells, multinucleated giant cells, and neoplastic stromal cells. It represents 4–5% of all primary bone tumors, primarily affecting individuals aged 20 to 45, with a slight female predominance. GCTB can behave as a benign, locally aggressive, malignant, or metastatic tumor. The stromal cells express nuclear factor kappa-Β ligand (RANKL), chemokines, and other cytokines that promote osteoclastogenesis [1,2].

Giant cell tumors of bone (GCTB) are mostly benign, with limited ability to metastasize to the lungs [3]. Metastasis occurs in a few patients, especially among those who have an aggressive and recurrent disease [4,5]. This tumor is metaphyseal and extends into the epiphyses of the long bones [6]. Inflammatory markers are increasingly being considered as playing a vital role in tumor growth, angiogenesis, invasion, and metastasis [7]. Overexpression of the receptor activator of the nuclear factor kappa-Β ligand (RANK) receptor is considered a hallmark of GCT, with the interaction of the RANK receptor and ligand found to be the culprit in massive bone resorption encountered with tumors [8].

Giant cells are multinucleated osteoclast cells, which are putative tumor cells of GCT. Various methods have been developed to grade the tumor according to its malignant behavior and recurrence, but they lack value. Enneking classification reflects only one aspect of aggressiveness, but the prediction of the tumor for metastasis and recurrence is non-existent. To date, there is no definitive histological, clinical, or radiographic criteria that have clearly predicted the behavior of giant cell tumor of bone (GCTB) in terms of outcomes [9].

In this study, the role of different genetic biomarkers is discussed, not only to diagnose GCTB but also for differentiating it from other tumors of bone containing giant cells, e.g., chondroblastoma, aneurysmal bone cyst, and giant cell tendon sheath tumor [10,11]. Also, to establish the implications of these genetic biomarkers to determine the local recurrence and metastasis of GCTB, as well as their significance in diagnosis of malignant GCTB. Some of the markers are upregulated with GCTB, e.g., VEGF, CD44, CD147, and P63, while others are downregulated, for example, miRNAs and Cx43 [12,13].

Every aspect of the pathways linked with GCTB is assessed. We summarize immunoglobulins, genetic mutations H3F3A, plasma proteins/amino acids, glycoproteins CD34, growth factors, and genetic patterns in this study [14,15]. This will help to understand the differentiation of tumors and predicting the prognosis of GCT. Future research should focus on exploring novel therapeutic targets related to the tumor’s biology and molecular mechanisms to improve clinical prognosis.

## 2. Methodology

**Figure 1:**
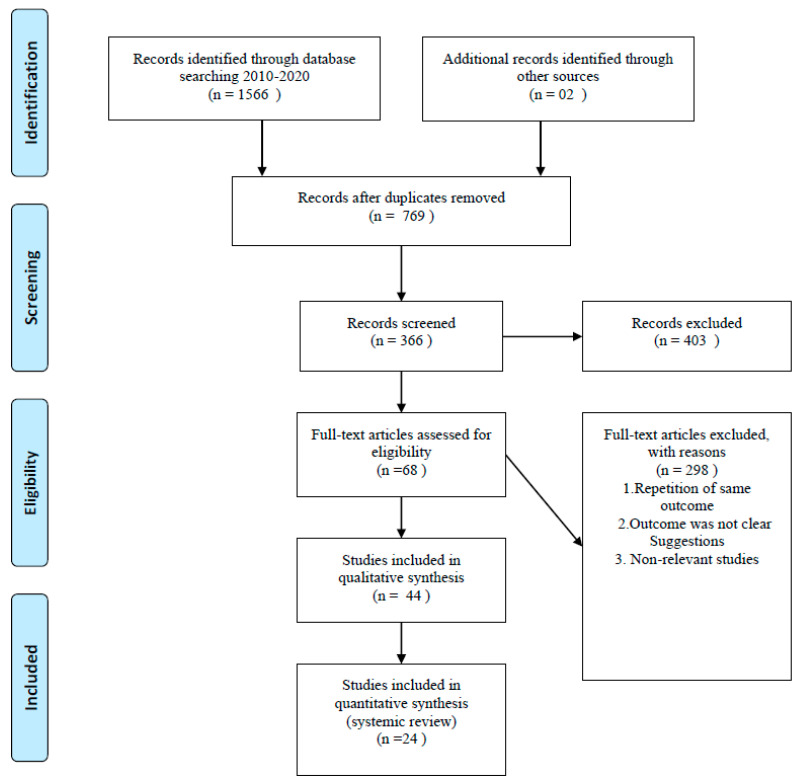
Prisma flow chart.

We conducted a literature search using Google, Google Scholar, PubMed, Wiley Library, Medline, Clinical trials.org, and Web of Science. Our search strategy included MeSH terms and keywords for giant cell tumor and biogenetic markers from January 2010 to September 2020. All three grades of the tumor, according to the Campanacci classification, were included in the study. All research articles in languages other than English were excluded. Phase I clinical trials, metanalyses, review articles, case reports, and case series were excluded from the study. After excluding review articles, duplicates, and non-relevant articles, we included 24 articles out of 1568 articles, summarizing the role of biogenetic markers in the prognosis of GCT as mentioned in Figure 1. We focused on the research question: Differentiation and identification of giant cell tumor from its malignant variant and other bony pathologies producing giant cells. As the primary endpoint of this review article is identification and differentiation of biogenetic markers, so these data will help to identify GCT, differentiation from other giant cell-containing pathologies, and prognosis of the disease.

## 3. Results

P63 is 98.6% sensitive and relatively specific for GCT as compared to other multinucleated giant cells containing neoplasms. MDM2, IGF1, STAT1, and RAC1 are associated with GCT recurrence, and so might serve as biomarkers for GCT recurrence. Immunofluorescent staining also indicated that CD29 and CD90 were all highly expressed in GCTB (giant cell tumor of bone) cells. Increased expression of the proteins STAT5B, GRB2, and OXSR1 was related to a higher probability of metastasis. Immunohistochemistry demonstrated that EMMPRIN (extracellular matrix metalloproteinase inducer) is present at the multinuclear osteoclast-like giant cells of GCT, with strong immunostaining on the cell membrane. H3F3A and H3F3B mutation analysis appears to be a highly specific, although less sensitive, diagnostic tool for the distinction of GCTB and chondroblastoma from other giant cells containing the tumor. The neutrophil-to-lymphocyte ratio (NLR) was ≤2.70 and > 2.70; the platelet-to-lymphocyte ratio (PLR) was ≤215.80 and >215.80; the lymphocyte-to-monocyte ratio (LMR) was ≤2.80 and >2.80; and the albumin-to-globulin ratio (AGR) was <1.50 and ≥1.50. Patients with an NLR > 2.70, PLR > 215.80, LMR ≤ 2.80, and AGR < 1.50 were significantly associated with decreased disease-free survival (DFS) (*p* < 0.05). Multivariate analysis indicated that treatment history, tumor length, bisphosphonate treatment, NLR, and PLR were independent factors of DFS (disease-free survival). Large amounts of osteoclast-related mRNA (cathepsin K, tartrate-resistant acid phosphatase, and matrix metalloproteinase9) in GCTs (*p* < 0.05) are associated with the grade of bone resorption. We propose that subarticular primary malignant bone sarcoma with H3.3 mutations represents true malignant GCTB, even in the absence of a benign GCTB component. IMP3 and IGF2 might be potential biomarkers for GCT of the spine in regulating the angiogenesis of giant cell tumor of bone and predicting patients’ prognosis.

## 4. Review Analysis

### 4.1. CD34

Over the years, clinical and radiographic criteria have been used to classify giant cell tumors into aggressive and non-aggressive types but these criteria cannot differentiate between the two types, posing a significant issue for the surgeons to define when surgical excision is deemed appropriate [16]. Dewsnup et al. reported that a glycoprotein, CD34, that acts as an intercellular adhesive channel in hematopoietic precursors and capillary endothelial cells has a high expression in aggressive tumors [17]. Susarla et al. retrieved histological samples of 32 patients between 1992 and 2006, with a preoperative diagnosis of isolated giant cell tumors [10]. A bivariate analysis was performed between CD34 expression density and giant cell tumor aggression, classified as any tumor with size >5 cm, recurrence, cortical bone thinning, or cortical bone perforation. CD34 staining density was described as a continuous binary measure: positive and negative. CD34 staining was performed after fixation in formalin, with monoclonal anti-CD34 antibodies, and developed with the modified avidin–biotin–peroxidase technique. The marker density was analyzed using BIOQUANT. Out of a total of 32 samples, 26 were preoperative for aggressive tumors and six were of the non-aggressive type. Using a *p*-value of <0.05, there was no statistically significant difference between both groups with regard to age and gender. Using the receiver operating characteristic (ROC) curve, it was demonstrated that CD34 staining density of ≥2.5% was a statistically significant threshold for aggressive tumors (CI: 1.6, *p* = 0.02). The threshold of ≥2.5% staining density had a sensitivity, specificity, positive predictive value, and negative predictive value of 0.75, 0.83, 0.95, and 0.45, respectively. In multivariate analysis, after controlling CD34 levels, age was found to be significantly negatively associated with tumor aggression, with each passing year being related to an 8% decrease in the chance of having an aggressive tumor with an odds ratio [OR] of 0.92 (95% CI: 0.85, 0.99, *p* 0.04), a result concurrent with the previously reported trials [18,19,20,21,22].

### 4.2. Plasma Metabolite Profiling

Genomics, transcriptomics, and proteomics provide a great understanding of complex biological systems, but their main focus of analysis is the upstream variations in genetics and proteins [23,24]. Metabolomics is an emerging field that describes a broad array of metabolic parameters that can be used along with the conventional protocols to aid in the diagnostics of GCT [25]. The changes in the tumor microenvironment can lead to significant variations in different metabolic pathways including glycolysis, lipolysis, proteolysis, the tricarboxylic acid cycle, and oxidative phosphorylation [24]. In the study by Wang et al. (2019), they compared 26 GCT patients with 28 healthy volunteers and mentioned a correlation coefficient [26,27]. All of the results were statistically significant (*p* < 0.05). The metabolites that were upregulated and demonstrated a positive correlation with GCT included glycerol (0.709), lactate (0.699), acetoacetate (0.499), O-acetyl-glycoprotein (0.477), acetone (0.44), 3-hydroxybutyrate (0.439), leucine (0.431), valine (0.353), isoleucine (0.348), and glutamate (0.334). The metabolites that were downregulated and had a negative correlation with GCT included betaine (−0.903), glycerophosphocholine (−0.873), formate (−0.835), glucose (−0.467), lysine (−0.399), methionine (−0.398), triglyceride (−0.334), and acetate (−0.318).

### 4.3. miRNA

MicroRNAs (miRNAs), small non-coding single-stranded molecules, have been shown to play an important role in tumor development. They negatively regulate their target genes and are involved in a broad spectrum of cellular functions, from cell growth and differentiation to cell apoptosis [28]. This is achieved by inhibiting translation or inducing mRNA degradation through binding to the 3′-untranslated region of their target mRNA [29,30,31]. The genes for miRNA are located in the fragile chromosomal regions. They can either function as tumor suppressors or oncogenes and, as a result, dysfunction at the level of miRNA is associated with tumorigenesis [32,33,34]. A study performed by Huang et al. has shown that there is a correlation between downregulated mi-R30 with GCT [35]. Another study, by Wu et al., has shown that miR-126-5p that suppresses matrix metalloproteinase (MMP) and affects osteoclast differentiation and bone resorption is downregulated in the spindle-like stromal cells of GCT [36]. Qin et al. studied eleven GCT patients with four controls and showed that expression of osteoclast-specific genes is increased in GCT. The genes that showed strong expression in GCT patients as compared to controls included cathepsin K, MMP-9, and TRAP. They also demonstrated that the expression of miRNA is different in GCT patients as compared to controls without GCT. Twenty miRNAs were studied using microarray data and six of them showed statistically significant decreased expression between cases and controls (*p* < 0.05). These six miRNAs included has-let7a-5p (*p* = 0.0001), has-mir-10b-5p (*p* < 0.0001), has-mir-16-5p (*p* = 0.0002), has-mir-106b-5p (*p* = 0.0055), has-mir-224-5p (*p* = 0.0001), and has-mir-876-5p (*p* < 0.01). In addition to this, the study also demonstrated that there is an association between the severity of bone resorption and miRNA expression. The two miRNAs that showed association with severity, measured by the Campanacci grading system, were has-mir-16-5p and has-let-7a-5p. Reduced expression was associated with severe bone resorption [37].

### 4.4. CD44, VEGF, and EZRIN

Cell adhesion molecules play an important part in tumor progression, and overexpression of various cell adhesion molecules is a hallmark for the prediction of tumor aggression. Ezrin is an abnormally expressed cell adhesion protein that affects cellular motility, apoptosis, proliferation, and thus tumor invasion and metastasis [38]. Cell adhesion factor CD44 has been associated with tumor progression, invasion, survival, metastasis, and tumor-free survival rates. VEGF, after binding to its specific receptor, is known to promote tumor proliferation, and after hydrolyzing the basement membrane, promotes vascular permeability. Zhang et al. collected 80 histological samples from Yunnan Provincial Tumor Hospital. Distant normal tissue from the same sample was used as a control. The immunophenotype of all three markers was identified by using immunohistochemical and Elivison staining. Using the chi-square test, a staining level less than 10% was considered as negative, the staining percentage, and clinicopathological factors were analyzed. A separate category of high expression was also analyzed separately, that had an expression ratio of ≥50%. It was found that Ezrin expression was 41.2% and 58.8% in GCT, respectively, for moderate and high expression rates, compared to 11.2% in adjacent normal tissue (χ^2^ = 39.670, *p* < 0.00). The CD44 high expression rate was 37.5% in GCT compared to 12.5% in adjacent normal tissue (χ^2^ = 13.333, *p* < 0.003). The VEGF high expression rate was 61.3% in GCT compared to 26.2% in adjacent normal tissue (χ^2^ = 19.911, *p* < 0.001). Spearman rank correlation analysis showed that the Ezrin and CD44 coefficient (r = 0.597, *p* < 0.001), Ezrin and VEGF coefficient (r = 0.741, *p* < 0.001), and VEGF and CD44 coefficient (r = 0.616, *p* < 0.001) were positively correlated. Tumor-free survival analysis showed survival was significantly lower for patients having Ezrin- (*p* ≤ 0.01), CD44- (*p* ≤ 0.01), and VEGF- (*p* ≤ 0.05) positive expression. These findings were consistent with similar findings noted in oesophageal, breast, ovarian, osteosarcoma, and liver tumors [39,40,41,42,43,44].

### 4.5. CD147 and PCNA, VEGF, and MMPs

EMMPRIN (CD147) is a member of the immunoglobulin superfamily, a cell surface glycoprotein. It has been found to be associated with tumorigenesis at various control levels [45,46]. Its capacity to degrade extracellular basal membranes and stromal elements through angiogenesis stimulates MMP production [47,48,49,50,51]. Yue Hu Han et al. collected 68 GCT samples at the Fourth Military University, China. All the samples were stained immunohistochemically using rabbit antihuman CD147 polyclonal antibody, mouse antihuman CD34 monoclonal antibody, rabbit antihuman VEGF monoclonal antibody, mouse antihuman PCNA monoclonal antibody, rabbit antihuman MMP-2 polyclonal antibody, and rabbit antihuman MMP-9 polyclonal antibody. A combined summed score from the percentage of cells stained and the intensity of staining was used to depict the association. It was found that 89.70% of samples were positive for CD147, and 61.76% had high expression. According to Spearmen’s rank correlation analysis, there was a significantly positive correlation between CD147 and MMP-9, VEGF, CD34, and PCNA (r = 0.271, *p* = 0.025; r = 0.411, *p* = 0.000; r = 0.872, *p* = 0.000; r = 0.394, *p* = 0.001).

### 4.6. P53 and P63 Gene Mutation

P53 is an ancestor gene, whose function is to guard any mutation in germline cell DNA. P63 has evolved subsequently, serving the same function in somatic cells, helping to maintain programmed proliferation of cells, halting any oncogenesis. P63 protein is encoded by the TP63 gene, located on chromosome 3q27-28TP [52]. We analyzed five studies with a total of 203 samples of giant cell tumors, out of which 142/161 (88.2%) showed acpositive P63 mutation, and 41/122 showed a positive p53 mutation. P63 and mutant p53 were detected using immunohistochemical testing with anti-p63 mouse monoclonal antibodies, electrophoresis on SDS-polyacrylamide gels, electroblotted to nitrocellulose membranes, and the western blot technique. Dickson et al., in their study, isolated mononuclear and giant cells from GCT using trypsin. Using 1:250 Dakocytomation, Mississauga, Canada, p63 anticolonial antibodies, they found that all tumors expressed p63 mutation, which was cultured later; and using a reverse transcription of RNA and PCR, clustered up to a TAp63 isoform of p63 [53]. A study by Hammas et al. concluded that all of the GCT cells tested positive for p63 expression. He also found that the sensitivity, specificity, positive predictive value, and negative predictive values for P63 immunohistochemistry were 100%, 74.42%, 59.62%, and 100% respectively, and all were statistically significant [54]. Yanagisawa et al., in their studies, put forth a p63-positive rate by calculating the percentage of cells which stained positive for p63 out of the total number of tumor cells. Calculations showed a 36.3% positive rate in non-recurrent tumors and a 73.6% positive rate in recurrent tumors, strengthening the p63 ratio as a good prognostic marker [55]. A study by Cheng et al. concluded that no significant statistical difference was observed in the local recurrence rate for mutant p53 and p63 gene expression [56]. Yalcinkaya et al. found a statistically significant relation between P53 expression and local recurrence in their study [57].

### 4.7. Cx43 Gap Junctions

Connexins, especially connexin43 and its membrane channel, are important regulators of osteoblast proliferation and differentiation, helping in the adaptation of osteocytes to mechanical loading and soluble growth factors [58]. Induced ablation of the GJA1 gene encoding connexion43 has been associated with osteoblast dysfunction and elevated osteoclast genesis. Similar pathological changes have been noted in neoplastic stromal cells of osteoblastic origin in giant cell tumors [59,60,61]. Peter et al. collected 131 surgically removed samples from the Laboratory of Experimental Oncology, Institute of Orthopedics Rizzoli, Bologna Italy, of which 89 were primary tumors and 34 were recurrent tumors. The tumors sectioned were then stained with rabbit anti-Cx43 antibodies. Additionally mononuclear and multinuclear giant cells were isolated with cell culture, FISH, reverse transcription, and western blot. The immune staining was semi-quantitatively measured using the 3DHISTECH software (Budapest, Hungary). In a semiquantitative analysis, it was found that Cx43 protein levels are statistically significantly reduced in osteoclast-rich tumor networks as compared to adjacent reactive stromal cells (*p* < 0.001). This relation was found for both Cx43 staining percentage and density. Additionally, no significant link between Cx43 and the frequency of GCTB recurrence could be elicited (*p* = 0.173). The FISH and cellular cultures were found clustered up to the Cx43 protein around the perinuclear area, suggesting an endoplasmic reticulum–Golgi body origin of the proteins in the stromal cells. Tumor-free survival was found to be associated positively with Cx43 expression using Spearman’s rank test.

### 4.8. Lumican (LUM) and Decorin (DCN)

Osteolytic and local aggressive characteristics of GCT are elicited by a high degree of expression of RANKL, that promotes osteoclast formation and their activity [62,63,64]. To plan appropriate surgical resection preoperatively effectively, we need to know a way to anticipate the clinical behavior of GCT [65,66,67]. Lieveld et al. collected 33 samples in total from the archives of the departments of Pathology of Leiden University Medical Center (The Netherlands) (n = 23), the N. Goormaghtigh Institute of Pathology of the Ghent University Hospital (Belgium) (n = 6), and the Rizolli Institute Bologna (Italy) (n = 4). In the samples, there were 24 cases of primary GCT, five had GCT with angiogenic bone cyst (ABC), and four patients had GCT with lung metastasis. Microarray analysis was used to analyze the differential expression of various proteins to rule out a null hypothesis or opt for an alternate hypothesis, to know about the differential expression of various proteins, which could be used as potential biomarkers for predicting GCT clinical behavior. Most stable housekeeping genes were identified using the Genome Housekeeping Gene Selection Kit. Q-PCR and immunohistochemistry were then utilized to confirm the relative gene expression. Analysis of the microarray result showed 11 genes that were significantly low in expression in the metastatic group. Of those 11 genes, Decorin and Luminan were the most differentially expressed. Luminan showed 30 times less expression (*p* value = 0.005), and Decorin was 26 (*p* value = 0.001) times less expressed. In recurrent GCT cases, it was found that Dermatopontin expression was lower and Zinc Finger Protein14 had a higher expression in the high recurrence group.

### 4.9. Ki-67

Ki-67, a nuclear protein, is commonly found in proliferating cells. It is seen in the proliferating phases of the cell cycle including the G1, S, G2, and M phases. It can serve as a marker of proliferation. It is not expressed in the resting G0 phase of the cell cycle. Due to the fact that Ki-67 indicates the proliferating cell percentage and not just the mitotic colonies, it can be correlated to the disease course as well. Yalcinkaya et al. 2015 mentioned that mononuclear cells and not the giant cells are responsible for the proliferative activity in the giant cell tumor of the bone, as evident in immunohistochemical staining studies. They also showed that the Ki-67 index was less than 10 percent in 3/10 (30%) of the recurrent cases and more than 10% in 7/10 (70%) of the recurrent cases [57]. Results from many studies have shown conflicting results about a higher Ki-67 proliferative index in recurrent tumors as compared to primary tumors [5,6,7,68]. Our review on a population of 153 patients with giant cell tumors shows that 31 recurrent tumors (20.3%) had a mean Ki-67 proliferative index of 16.831 in 122 cases, with non-recurrent tumors (79.7%) having a mean index of 9.26. Although, studies by Cheng et al. and Ismail et al. 2010 did not independently show a statistically significant association between Ki-67 and recurrence [69]. Ismail et al. demonstrated that the Ki-67 index mean value was 6.68 in patients with pulmonary metastasis as compared to 2.89 in patients without metastasis [70]. Ki-67 has a short half-life. This cell percentage is reflective of the Ki-67 proliferative index. As suggested, the proliferative index is higher in aggressive tumors, and hence the results can be extrapolated to correlate with prognosis as well [70,71,72].

### 4.10. IGF1, STAT1, RAC1, and MDM2

Giant cell tumor has a variable behavior pattern, ranging from benign to malignant and recurrent tumors, leading to difficulty in evaluation and surgical planning [73]. Among proposed biomarkers for predicting GCT behavior are the following: mouse double minute 2 homolog (MDM2), insulin-like growth factor 1 (IGF1), signal transducer and activator of transcription 1 (STAT1), and Rac family small GTPase 1 (RAC1), which are involved in tumor progression. MDM2, an oncogene regulating P53, amplification can lead to the deregulation of the cell cycle and inhibition of apoptosis, resulting in a number of malignancies [74,75]. IGF1 has a key role in osteoblast and chondroblast proliferation, differentiation, and survival. It is directly related to bone mineralization and growth [76]. STAT1 is a member of the transcription factors; it has a pro-apoptotic and anti-proliferative effect. It is involved in many human malignancies like breast cancer, melanoma, leukemia, lymphoma, and other solid cancers [77]. RAC1 is a GTPase signaling protein, regulating glucose uptake, cell growth, and cytoskeletal reorganization. It regulates tumor invasion, metastasis, and angiogenesis [78]. According to a cohort study, Shuxin Chen et al. compared 55 recurrent vs. 20 non-recurrent GCT patients with ages ranging from 15 to 65 years. Immunohistochemistry and statistical analyses were conducted to confirm the association between the expression of the four genes and GCT recurrence. The expression of MDM2, IGF1, STAT1, and RAC1 was significantly higher in the recurrent group than that in GCT patients without recurrence (*p* < 005). However, the expression of these genetic biomarkers was not associated with age, gender, Campanacci grade, pathological fracture, or lung metastasis. MDM2, IGF1, STAT1, and RAC1 may be used as valuable biomarkers to predict GCT recurrence.

### 4.11. PRX1, GPX1, UBE2N, and AIF1

Giant cell tumor is locally aggressive and has metastatic potential to the lungs. Several antioxidants play a very important role in cell protection and cell remodeling. Glutathione peroxidase-1 (GPX1) is a member of the phylogenetic enzymes; it interacts with oxidative stress and takes part in several important biological systems. In the initiation phase, it protects the cell from oxidative stress but its overexpression may lead to malignancies [79]. Peroxiredoxins (Prxs) are peroxidases that scavenge H_2_O_2_ and provide protection from free radicals. The exact mechanism of their contribution towards tumorigenesis is unknown [80]. Similarly, Ubiquitin-Conjugating Enzyme E2N (UBE2N) regulates cell cycle progression, inflammatory markers, and the mechanisms through which they attune cancer metastasis [81]. Heat shock protein 27 (Hsp27) is a multidirectional protein that functions as a protein chaperon and antioxidant. These are induced by environmental stressors and heat shock [82]. Amalia et al. conducted a cohort study on 155 patients, emphasizing the role of antioxidants in tumorigenesis. They explained that genes of PRX1, GPX1, UBE2N, and AIF1 proteins were 1.4 to 3 times increased in metastatic disease while Hsp27 and lysozyme mutant T11A were downregulated with a significant *p*-value. In the non-metastatic group, expression of GPX1 and A1F1 was weak or undetectable. After a follow-up of 60 months, overexpression in the locally relapsed group compared with the disease-free group was as follows: GPX1: 71% versus 24%, χ^2^ = 35.04, *p* = 0.0001; PRX: 65% versus 54%, *p* = NS; and AIF1: 63% versus 36%, χ^2^ = 15.0, *p* = 0.03. 

### 4.12. Proteins STAT5B, GRB2, and OXSR1

Tumor cells have a proteolytic activity that produces biomarkers that circulate in the serum. These biomarkers can be used for diagnosis as well as prognosis of tumor [83]. These biomarkers mainly include the low-molecular-weight proteins and their fragments that have shown an association with various disease states including cancers and infectious pathologies, as well as metabolic diseases [84,85,86]. Coni et al. studied 924 low-molecular-weight proteins and protein fragments using a nanoparticle technique and mass spectrometry analysis in 20 GCT patients and described 43 proteins that were newly expressed in the GCT patient population [12]. In addition to the de novo expressed proteins, twenty-four proteins that were variably abundant in the GCT patients as compared to healthy controls were also observed. Out of these twenty-four, the ones with more than a five times fold change (*p* < 0.05) included alpha-enolase (16.0779), vitamin D-binding protein (14.13), signal transducer and activator of _transcription_5B (9.10), insulin-like growth factor-binding protein 7 (7.12), coagulation factor XI 1.15 0.03 0.044 (6.31), growth factor receptor-bound protein-2 (6.20), and serum amyloid A-4 protein (5.52). Conti et al. also showed a statistically significant difference (*p* < 0.05) in twenty proteins when compared between metastatic and non-metastatic patients. The most significant of these proteins upregulated in the metastatic patients concerning the fold change included oxidative-stress responsive 1 protein OXSR1 (17.50), RAB11A (14.54), and STAT5B (12.00). Proteins that were downregulated in the metastatic samples and showed higher concentrations in non-metastatic patients included TUBB1 (−14.10) and FLNA (−30.60) [87].

### 4.13. Inflammatory Biomarkers and Progression of GCT

In the interest of finding potential biomarkers and their biological behavior in the progression of the disease, previous studies have established an association between the expression of biological markers such as insulin-like growth factor 2 (IGF-2), insulin-like growth factor 2 mRNA-binding protein 3 (IMP-3), inflammatory biomarkers, and interleukin-17A (IL-17A) with the progression and recurrence of giant cell tumors of the bone [88]. Study results presented by Zhang et al. revealed a significant association between the expression of IGF-2 and IMP-3 with the progression of GCT in the form of tumor extension and recurrence, regardless of the patient’s age, gender, tumor location, and size of the tumor (*p* < 0.05). Overexpression of both IMP-3 and IGF-2 was claimed to have a critical role synergistically in promoting tumor angiogenesis, as out of 38 samples of giant cell tumors, 34.2% (13/38) and 44.7% (17/38) showed IMP-3 and IGF-2 expression and were significantly correlated with tumor microvascular density (*p* < 0.001 and *p* 0.005), respectively. For determination of prognostic factors other than the tumor characteristics, Jialin et al., in their study, revealed that the host’s inflammatory response to the tumor, as recognized by tumor-infiltrating inflammatory cells, which further stimulates the production of inflammatory mediators and cytokines, resulting in angiogenesis of the tumor, promoting its growth, metastasis, and invasion, may play a role as a parameter in predicting the prognosis of patients with giant cell tumors of the bone [89]. The combination of these factors and their indices, such as the neutrophil-to-lymphocyte ratio (NLR), platelet-to-lymphocyte ratio (PLR), lymphocyte-to-monocyte ratio (LMR), and albumin-to-globulin ratio (AGR), were analyzed after gross total resection of pathologically proven tumor samples from a total of 129 patients with giant cell tumor of the spine via nomograms to predict the disease-free survival (DFS) in these patients. To show the correlation of these indices with an increase or decrease in the disease-free survival, the patients were stratified into two groups: those showing an NLR > 2.70, PLR > 215.80, LMR ≤ 2.80, and AGR < 1.50 were significantly associated with decreased DFS (*p* < 0.05).

Another tumor marker that may contribute to the progression of GCTB is the overexpression of IL-17A, as supported by Meng Xu, et al. in their study results, which might be a potential biomarker in determining the progression and also as a therapeutic target in the treatment of giant cell tumor of the bone [89].

### 4.14. H3F3A and H3F3B

The H3F3A and H3F3B (H3 histone family member 3A and 3B) genes encode a protein called Histone H3.3, which plays an important role in maintaining the integrity of the genome during mammalian development. H3F3A gene mutation (G34W/V/R/L) has been found to be the most common mutation in giant cell tumors of the bone. We analyzed four studies with a total of 345 samples of giant cell tumors, out of which 300 (86.95%) showed H3F3A mutation, using immunohistochemical testing with anti-histone 3.3 (G34W) monoclonal antibodies, and direct sequencing with polymerase chain reaction (PCR) and co-amplification at lower denaturation temperature. Amary et al. and Schaefer et al., in their studies, used immunohistochemical staining with monoclonal antibodies against Histone H3.3 G34W, which revealed a strong nuclear expression of H3F3A mutation in 235/261 (90%) of the GCTB samples included in their studies, evidencing it as a reliable marker for detecting H3F3A gene variation in GCTBs [90]. Cleven et al., from their study, concluded that the expression of trimethylated Histone H3K36 protein being observed in the 59/60 (98%) GCTB samples, 41 (69%) out of which were positive for H3F3A gene mutation, suggests that methylation at this particular residue may play a role in the etiology of the disease as it showed a correlation with H3F3A gene mutation, but it cannot be used as a surrogate marker for H3F3A mutation status [91].

## 5. Discussion

Giant cell tumors are benign osteolytic tumors with limited metastatic capability. Surgical treatment for the tumor is based upon Campanacci staging of the tumor, which may range from en bloc resection to extensive bone dissection, intramedullary drilling, and chemical cauterization. The aggressiveness of the tumor is also an important factor in defining the role of chemotherapy and the extent of surgery. With a limited potential to metastasis (1–9%), giant cell tumors can have an excellent prognosis and the stress of repeated surgeries can be avoided if the aggressiveness of the tumor can be predicted in the preoperative period. Biochemical and immunohistochemical markers have over the years proved their vital roles in the management of various tumors and help to decide the extent of treatment deemed in each case. This review article evaluates the effectiveness of various biochemical criteria and immunohistochemical markers to predict the aggressiveness, tumor-free survival duration, and chances of metastasis in the preoperative period by predicting tumor behavior as summarized in Table 1.

CD34 was found to be a significant staining marker, with a threshold staining level of ≥2.5%, and had a specificity of 83% and a positive predictive value of 95%, with no statistically significant difference concerning age or gender. Similarly, it was also noted that GCT cells were positive for expression of p63 at a percentage of 88.2%, with a sensitivity of 100%, and a negative predictive value of 100% [100,101]. GCT cells were found to have a positive expression rate of 89.7% for CD 147 and 61.76% had a high expression for CD147/Ezrin, at a level of 10% staining as positive, and had an expression percentage of 41.2% for moderate staining and a 58.8% positive rate for high expression. VEGF had an expression of 61.3% in GCT cells, as compared to 26.2% in adjacent cells. Gelatin zymography showed that metalloproteinase contributes to proteolysis, which results in vascular invasion and local bone resorption [102]. CD44 had an expression rate of 37.5% as compared to surrounding normal tissues. It has been suggested that the recurrence of the tumor is related to the Campanacci grade, but this was later refuted by other studies [103]. Ki-67 was found to have an elevated expression in cases of recurrent tumors (16.831%) as compared to primary tumors (9.26%). Similarly, Ki-67 levels were also high in cases of pulmonary metastasis. Gene expression rates of IGF1, STAT1, and RAC1 were found to be higher in cases of recurrent tumors than in primary tumors, statistically significant enough to predict recurrence of GCT. The expression of various antioxidants, including PRX1, GPX1, UBE2N, and AIF1, was significantly higher in metastatic tumors. In another study, Midori Toda described GPX1 as a maker of aggressiveness [104]. Taketo Okubo narrated that overexpression of GPX1 leads to aggressiveness, metastasis, and recurrence [104]. Nanoparticle analysis using mass spectrometry found fivefold changes in the expression levels of alpha-enolase, vitamin D-binding proteins, signal transducer and activator of transcription, insulin-like growth factor-binding proteins, and the serum amyloid A-4 protein. Connexion43 had a decreased staining intensity for the GCT cells. CD34 immune staining was found to be a significant diagnostic criterion for GCT, while p63 expression can be of benefit for screening for GCT samples. Ibrahim et al. described the role of P63 in GCT differentiation from other tumors [105]. In another study, bivariate analyses yielded a mean sensitivity of 0.87 (95% confidence interval) and specificity of 0.71 (95% CI) for p63, which can be used as a biomarker in diagnosing GCTB [102].

Correlation studies have shown an increase in levels of glycerol (0.709), lactate (0.699), acetoacetate (0.499), O-acetyl-glycoprotein (0.477), acetone (0.44), and 3-hydroxybutyrate (0.439); a negative correlation was found for GCT with betaine (−0.903), glycerophosphocholine (−0.873), formate (−0.835), and glucose (−0.467).

The limitation of this study is that it is a review paper including a limited number of studies and most of them are retrospective studies. There has been no integration of GCT tumor markers into a preoperative assessment tool. Such markers can predict tumor behavior in advance, allowing for a meticulous approach, e.g., denosumab, radiotherapy, and extended curettage. In the future, to clinically prove the value of prognostic and diagnostic markers, a randomized clinical trial with a large sample size, representing the population size, is recommended to predict the aggressiveness and chances of metastasis in GCT. New tumor markers need to be investigated for a better understanding of and treatment of giant cell tumors. Along with the growing knowledge about tumor anatomy, physiology, and pathogenesis, several researchers are devoting themselves to try to find other ways to predict the nature and behavior of tumors to decrease the morbidity and mortality of giant cell tumor.

## 6. Conclusions

To date, histology shows a mixed picture of giant cells, making the task of giant cell tumor one of differential diagnosis. Some tumors metastasize while others stay benign. In many cases, the treatment of GCT is followed by recurrence. To answer these questions, some biogenetic markers need to be evaluated and assessed to predict the malignant nature and recurrence of the tumor, so that a definitive treatment plan can be opted for such cases. There is a need to include these biogenetic markers in the routine work-up for definitive diagnosis and effective management of giant cell tumor. This study has shown serological markers, genetic factors, cell membrane receptor markers, predictive markers for malignancy, and prognostic protein markers which are highly sensitive for GCT and relatively specific for giant cell tumor. MDM2, IGF1, STAT1, RAC1 are important markers in determining recurrence, while P63 and H3F3A differentiate GCT from other giant cell-containing tumors. STAT5B, GRB2, and OXSR1 are significant in determining the prognosis of GCT. Apart from using radiological and histological parameters, we can add them to tumor work-up for definitive diagnosis and prognosis.

## Figures and Tables

**Table 1:** Significance of biogenetic markers in giant cell tumor differentiation and prognosis.

Author	Name of Biomarker	Type of Study	Age Years	Sample Size	Gender	Comparison Groups	Results
Susarla et al. [10]	CD34	Retrospective study	24.4 ± 19.4	32	9M/23F	Aggressive (26) vs. non-aggressive (6)	CD34 staining density of ≥2.5% staining density had a sensitivity, specificity, positive predictive value, and negative predictive value of 0.75, 0.83, 0.95, and 0.45, respectively.
Jing Zhang et al. [11]	Ezrin, CD44, VEGF	Cross-sectional	14–57	80	45M/35F	GCT vs. normal tissue	Expression status of ezrin, CD44, and VEGF were significantly higher for GCTB at Campanacci stage III than at Campanacci stages I and II (*p* < 0.05).
Amalia et al. [13]	GPX1, PRX, AIF1, UBE2N, Hsp27	Cohort study	NA	155	NA	Pulmonary metastatic vs. non-metastatic	High expression of glutathione peroxidase 1 was strongly related to local recurrence and metastasis.
Wei Wang et al. [23]	Plasma metabolites	Case control study	27–51	Case = 28 Control = 26	19M/35F	NA	Metabolomics profiling approach is a promising screening tool for the diagnosis and relapse monitoring values for GCT.
Shu Qin et al. [37]	miRNA	Clinical article	17–61	Case = 11 Control = 4	5M/6F	GCT vs. normal tissue	Downregulated has-mir-16-5p and has-let-7a-5p expression indicate severe bone destruction.
Hammas et al. [54]	P63	Retrospective study	8–59	48	26M/22F	NA	The sensitivity and negative predictive value (NPV) of P63 is 100%. The specificity and positive predictive value (PPV) are 74.42% and 59.26%, respectively
YANAGISAWA et al. [55]	P63	Retrospective	NA	39	NA	Recurrent and non-recurrent	The mean p63-positive rate for recurrent GCT cases (73.6%) was statistically higher than that for non-recurrent cases (29.1%). P63 appeared to indicate the biological aggressiveness of GCT.
Dong-dong Chenga et al. [56]	Ki-67	Cohort study	NA	80	49M/31F	Recurrence vs. without recurrence	Ki-67 and CD147 expression, pathological fracture, Campanacci grade, and surgical method were associated with recurrence.
Yalcinkaya et al. [57]	p53 Ki-67	Retrospective	20–40	42	20M/22F	NA	A statistically significant relationship was found between p53 positivity, local recurrence, and lung metastases.
Faisham W Ismail [69]	Ki-67	Retrospective study	33.8		19M/12F	NA	Ki-67 index is not useful prognostic marker for aggressive type of giant cell tumor of the bone.
Zhang et al. [88]	IMP3 and IGF2	Cross-sectional study	32.6	38	15M/23F	NA	Positive expression of IMP3 and IGF2 was tightly related to the tumor extension and local recurrence of GCT.
MengXu et al. [89]	IL-17A	In vitro	16–58	74	33M/41F	NA	IL-17A is more frequently over expressed in aggressive lesions.
Jialin Li et al. [90]	Inflammatory biomarkers	Retrospective study	11-69	129	55M/74F	NLR ≤ 2.70 and >2.70; PLR ≤ 215.80 and >215.80; LMR ≤ 2.80 and >2.80; AGR < 1.50 and ≥1.50	NLR > 2.70, PLR > 215.80, LMR ≤ 2.80, and AGR < 1.50 were significantly associated with decreased DFS (*p* < 0.05).
Arjen et al. [91]	H3F3A, H3F3B	In vitro study	27–35	72	46M/36F	Giant cell vs. chondroblastoma	The majority (69% of GCTBs harbored an H3F3A G34W/V mutation, whereas mutations were absent in other giant cell-containing tumors. H3F3B K36M mutation is present only in chondroblastoma.
Gustavo et al. [92]	P63	Retrospective	NA	80	NA	NA	The sensitivity, specificity, PPV, and NPP of p63 were 86.95%, 53.36%, 45.45%, and 91.17%, respectively
Shooshtarizadeh et al. [93]	P63 (member of P53)	Cross-sectional	11–62	100	NA	NA	GCT is 96.8% positive for P63. P63 is 98.6% sensitive and relatively specific for GCT.
M. Lieveld et al. [94]	Lumican (LUM) and decorin (DCN)	Retrospective study	15–51	74	31M/43F	Metastatic vs. non-metastatic	Decorin and luminan were the most differentially expressed. Luminan showed 30 times less expression (*p* value = 0.005), and decorin was 26 (*p* value = 0.001) times less expressed in metastatic group.
Shuxin et al. [95]	MDM2, IGF1, STAT1, RAC1	Cohort study	15–65	75	36M/39F	Recurrence vs. non-recurrence	Expression of MDM2, IGF1, STAT1, and RAC1 in GCT patients with recurrence was significantly higher than that in GCT patients without recurrence (*p* < 005).
Fernanda Amary et al. [96]	H3.3 G34W	Cross-sectional study		3163		NA	Subarticular primary malignant bone sarcoma with H3.3 mutations represent true malignant GCTB, even in the absence of a benign GCTB component.
Marie Schaefer et al. [97]	H3F3A and H3F3B	In Vitro	13–84	55	24M/32F	NA	H3G34W and H3K36M IHC is highly specific for GCT and chondroblastoma, respectively.
Alberto Righi et al. [98]	Histone 3.3 variant of H3F3A	In vitro		68		NA	96% of giant cell tumor of bone harbored an H3F3A mutation. Secondary malignant giant cell tumors also presented an H3F3A mutation.
Peter Balla et al. [99]	Cx43	Cohort	32.46	123	70M/53F	NA	Downregulation of Cx43 expression and gap junction coupling in neoplastic stromal cells are associated with the clinical progression and worse prognosis in GCTB.

## Data Availability

The raw data supporting the conclusions of this article will be made available by the authors on request.
